# Novel Design Scheme for Structural Fundamental Frequency of Porous Acoustic Metamaterials

**DOI:** 10.3390/ma15196569

**Published:** 2022-09-22

**Authors:** Ying Zhou, Hao Li, Mengli Ye, Yun Shi, Liang Gao

**Affiliations:** 1 State Key Lab of Digital Manufacturing Equipment and Technology, Huazhong University of Science and Technology, 1037 Luoyu Road, Wuhan 430074, China; 2 Shanghai Aerospace Equipments Manufacturer Co., Ltd., 100 Huaning Road, Shanghai 200245, China

**Keywords:** acoustic-structure interaction system, full-cycle interactive progressive design, surrogate model, frequency modulation acoustic metamaterial

## Abstract

Structural resonance increases the vibration and noise of porous acoustic metamaterials while reducing the energy consumption and conversion efficiency of acoustic waves. Therefore, structural fundamental frequency of porous acoustic metamaterials is required to be controlled to avoid resonance. This study proposes a full-cycle interactive progressive (FIP) design scheme for porous acoustic metamaterials. The FIP design scheme first establishes a specific parameter relationship for the initial model based on the intentions of the designers. The initial model is then dynamically adjusted through a series of optimization processes. In particular, the FIP design scheme is developed for a porous acoustic metamaterial in an acoustic-structure interaction system. The effects of the structural parameters and applied boundary conditions of the porous acoustic metamaterial on the structural fundamental frequency are investigated. A surrogate model is introduced to reduce the calculation costs and improve the design efficiency of the parametric optimization. The frequency-modulation acoustic metamaterial is tailored to improve its acoustic and vibrational characteristics, including the resonance resistance and low dynamic response. The features of the FIP design scheme in the optimized design of porous acoustic metamaterials are demonstrated.

## 1. Introduction

Vibration and noise are correlated with each other and have become important factors in the manufacturing industry, affecting product quality, reducing operational accuracy, and shortening product life [1,2]. Modal control of acoustic structures is an indispensable task for avoiding resonance and reducing noise. There are several modal control schemes for porous acoustic structures. Among them, the structural fundamental frequency is widely used for its simple modeling, efficient solution, and stable optimization. From the structural design perspective, the fundamental frequency of porous acoustic structures according to practical working conditions can be optimized and adjusted to avoid resonance, reduce noise, and improve the stability of the acoustic system [3]. This study aims to raise the structural fundamental frequency by devising a unique type of porous acoustic metamaterial. Metamaterials are a type of artificial materials/structures involving novel geometric shapes and extraordinary physical properties [4,5]. The macroproperties of metamaterials significantly depend on the innovative design structure rather than the physical properties of the constituent materials [6,7]. The frequency-response analyses of different acoustic metamaterials have been extensively studied by various scholars. Various frequency response design models for acoustic–structure interaction (ASI) systems were established by Yoon et al. [8] and Vicente et al. [9] to obtain the corresponding acoustic metamaterial. The boundary interface description, coupling interface assignment, and local modal problems were also solved in these design processes. Moreover, the vibration and noise reduction effects of these optimized structures in practical applications were verified via multiple engineering cases, illustrating the necessity of frequency response analysis for acoustic metamaterials. In this study, a frequency modulation acoustic metamaterial (FMAM), which is a porous acoustic metamaterial with better vibration and noise reduction performance, was designed to reduce the level of structural response and avoid resonance.

It is difficult to complete a complex model description of porous acoustic metamaterials using conventional acoustic materials. Therefore, this study improves the traditional optimization design method and proposes a full-cycle interactive progressive (FIP) design scheme for porous acoustic metamaterials [10,11]. FIP design is a full-cycle optimization method that seeks the optimal design in a gradient and interactively adjusts the design direction [12]. It includes three parts: topology optimization based on the ASI system, parametric optimization based on the surrogate model (SM), and experimental analysis of the frequency response based on the closed acoustic box. An ASI system-based topology optimization provides an innovative initial structure for the FIP scheme of porous acoustic metamaterials [13,14]. Topology optimization is a powerful tool to optimize the material distribution within a given design domain, so as to achieve the best structure performance under some design constraints. There are many methods that have been established for topology optimization, e.g., the homogenization method [15,16], solid isotropic material with penalization (SIMP) [17,18,19], evolutionary structural optimization (ESO) [20], level set method (LSM) [21,22,23,24], and so on. Most of the topology optimization formulations can be efficiently solved by gradient-based algorithms [25,26,27] or intelligent optimization algorithms [28,29].

Structural deformation in an ASI system is generally non-linear, while a linear deformation is an ideal situation. However, there is insufficient research on topology optimization based on non-linear finite elements, mainly due to the lack of optimization methods and calculation power. Recently, with the rapid development of hardware computing capabilities, an increasing number of scholars have extended traditional topology optimization methods to non-linear fields [30,31]. Singh et al. [32] observed non-linear acoustics to have the potential to identify damages in composite structures that are difficult to detect using conventional linear ultrasonic methods and presented a rapid approach to model the non-linear behavior caused by closed delamination. Additionally, a few parametric studies have been performed to investigate the effects of various parameters related to the non-linear phenomenon. Some experiments were conducted by Zhang et al. [33] with a focused transducer working in pulse-echo mode, and the measured amplitudes of non-linear waves reflected upon water–air and water–aluminum/steel interfaces conformed with the simulated results to help advance applications using pulse-echo non-linear acoustics.

Parametric optimization based on the SM includes the core stage of the FIP design. The SM is a data-driven analysis model that can approximate the implicit relationship between design variables, objective functions, and constraints [34]. It can effectively reduce the calculation costs and improve the design efficiency of parametric optimization. The calculation result by using SMs is recognized to have a very high accuracy [35,36]. Extensive investigation of SMs has enabled prevalent use of this model in actual engineering optimization designs. Li et al. [37] proposed a multisampling point sequence global optimization algorithm based on the Kriging model, whose numerical and simulation examples verify its effectiveness and practicability. Topology and parametric optimizations with the application of response surface method was implemented for the optimal design of a jig reinforcement structure by Cho et al. [38] to minimize the total jig weight while securing the dimensional precision of a foamed urethane case. Lin et al. [39] proposed two-stage artificial neural network-based hole image interpretation techniques to develop a fully automated configuration optimization system with improved template variety and recognition reliability.

The experimental analysis of the frequency response based on the closed acoustic box includes a verification analysis of the FIP design. It is also necessary to ensure the effectiveness of the optimization results. This process conducts a simulation analysis under practical working conditions and avoids excessive simulation and application deviations. Moreover, the porous acoustic metamaterial unit cell obtained using parametric optimization is distinguished by its complex structure and small macroscopic size. Traditional processing methods are unable to satisfy manufacturing requirements. Based on the additive manufacturing method, our method realizes high-precision manufacturing of optimized structures while the size and shape accuracy satisfy the subsequent experimental requirements.

This study proposes an FIP design method for the fundamental frequency maximization of an FMAM structure. FIP design method can seek the optimal design in a gradient and interactively adjust the design direction to complete a complex model description of porous acoustic metamaterials. FMAM is designed to improve the acoustic and vibration characteristics, avoid resonance, and reduce the level of structural response of acoustic metamaterials effectively. Modal acoustic characteristics of FMAM are studied through a closed acoustic box based on the ASI system. The remainder of this paper is organized as follows: Section 2 introduces the application background and theories of the frequency modulation process of FMAM. Section 3 completes the topology optimization with the design goal of maximizing the fundamental frequency and obtaining the initial FMAM configuration. Section 4 establishes the three-dimensional FMAM model based on the above-mentioned two-dimensional initial configuration and performs parametric optimization based on the SM. Moreover, feedback adjustments were performed to strengthen the parametric optimization effect. Section 5 describes the construction of a closed ASI box to study the modal and acoustic characteristics of FMAM. Section 6 summarizes the content of this study. All acronyms are given in the Abbreviations.

## 2. Research Problems on FMAM

Noise is a primary form of external excitation in acoustic structures. To ensure a healthy and effective working environment, acoustic insulation and noise reduction materials are worth researching. In particular, if the frequency of an external excitation is close to the natural frequency of the acoustic structure, the amplitude of the acoustic structure increases sharply. Strong vibrations inevitably lead to the aggravation of noise, affecting the overall vibration and noise reduction performance of the acoustic structure.

In this study, FMAM was designed using a straightforward optimization design method to avoid resonance. In previous studies, the shape or size of some structures was optimized to change their natural frequency while they were bound to a single initial structure. The rigidity and mass distribution of some structures were improved to change their natural frequency; however, it was difficult to satisfy the practical requirements for high-dimensional complex structures [40,41,42]. Considering the characteristics of the previous design methods, the FIP design method was used in this study to investigate the natural frequency of acoustic metamaterials.

There are several objective functions for the FIP design method to control the natural frequency of porous acoustic metamaterials such as maximizing the fundamental frequency, minimizing the difference between the natural frequency of any specified order and a given frequency, and maximizing the gap between adjacent natural frequencies. Among the above optimization objectives of the natural frequency, the optimization model with the maximum fundamental frequency was adopted because of its convenience such as stable iteration and rapid convergence. Therefore, maximization of the fundamental frequency of the porous acoustic metamaterial is used as the objective function in the FIP design method.

When a plane wave with finite amplitude propagates in an ideal fluid medium with no viscous loss, the motion and continuity equations of a non-linear medium considering the one-dimensional propagation along the x direction are expressed, respectively, as follows [43]:

(1) $$\frac{\partial v}{\partial t}+v\frac{\partial v}{\partial x}=-\frac{1}{\rho }\frac{\partial p}{\partial x},$$

(2) $$\frac{\partial (\rho v)}{\partial x}=-\frac{\partial \rho }{\partial t},$$

where v represents the particle velocity, which can be obtained from the acoustic wave power, p denotes the acoustic pressure, and ρ indicates the medium density. At this instant, the non-linear term $v\frac{\partial v}{\partial x}$ has almost the same order of magnitude as the other terms.

In an ideal medium, the particle velocity v=v(ρ) and sound velocity c=c(ρ) are both single-valued functions of density ρ for adiabatic processes. Substituting these into Equations (1) and (2), we obtain

(3) $$\left\{\begin{array}{l} \frac{\frac{\partial v}{\partial t}}{\frac{\partial v}{\partial x}}=v+\frac{1}{\rho }(\frac{dp}{dv})\\ -\frac{\left(\frac{\partial \rho }{\partial t}\right)}{\left(\frac{\partial \rho }{\partial x}\right)}=v+\rho (\frac{dv}{d\rho }) \end{array}\right.$$

The left-hand side of the above formula represents v, which is the derivative of the constant ${\left(\frac{\partial x}{\partial t}\right)}_{v}$, and ρ is the derivative of the constant ${\left(\frac{\partial x}{\partial t}\right)}_{\rho }$. However, the value of v can only be determined by ρ. Regardless of whether the value is constant for ρ or v, there is no difference in the derivative of t. So:

(4) $${\left(\frac{\partial x}{\partial t}\right)}_{v}={\left(\frac{\partial x}{\partial t}\right)}_{\rho }$$

From Equation (3), we can get:

(5) $$\left\{\begin{array}{l} \rho^{2}(\frac{dv}{d\rho })=\frac{dp}{dv}=c^{2}(\frac{d\rho }{dv})\\ {\left(\frac{dv}{d\rho }\right)}^{2}=\frac{c^{2}}{\rho^{2}} \end{array}\right.$$

where c is the acoustic speed and $c^{2}=\frac{dp}{d\rho }$. From Equation (5), we obtain

(6) $$v=\pm \int \frac{c}{\rho }d\rho =\pm \int \frac{dp}{\rho c}$$

Using this formula, the general relationship between the particle velocity v, density increment $\rho^{\prime }$, and acoustic pressure p can be determined. Integrating Equation (6) into p, we obtain

(7) $$p=p_{0}\cdot {\left(1\pm \frac{(\rho_{0}c_{0}^{2}-p_{0})c^{2}\rho }{2pp_{0}c_{0}}\right)}^{\frac{2v}{1\pm v}}$$

where p represents the total pressure generated by the acoustic wave motion and $p_{0}$ denotes the maximum value of the pressure changes, i.e., the maximum value of the acoustic pressure. For plane continuous sound waves, the external sound pressure matrix p on the ASI interface can be obtained using Equation (7). According to the above-mentioned equations, the steady-state boundary equations of the acoustic pressure and displacement at the boundary nodes of the acoustic field are as follows:

(8) φp=Gu−Hp

On the interface $\Gamma_{s}$ between the solid structure and air, the external acoustic pressure matrix p and solid internal elastic force matrix F satisfy the balance equation of the coupling interface, which is expressed as follows:

(9) $$F\cdot n_{s}=p\cdot n_{a}$$

where $n_{a}$ and $n_{s}$ represent the normal vectors of air and the solid structures acting on the coupling interface, respectively. From the stiffness matrix, mass matrix, and elastic constitutive relationship, the equilibrium equation of the ASI model can be obtained as follows:

(10) $$F=(K-\omega^{2}M)u$$

where F, K, M, and u represent the total pressure, overall stiffness matrix, and overall displacement matrix, respectively, generated by the large-amplitude acoustic wave motion of the optimized model. Equation (10) is an implicit equation of the structural natural frequency ω, and is the main constraint condition for subsequent topology optimization. For a continuous structure system with *n* degrees of freedom, *n* continuous natural frequencies exist. Among them, the low-order natural frequency, particularly the first-order natural frequency (i.e., the fundamental frequency), is highly significant for analyzing the dynamic characteristics of the structure.

Owing to the non-linear characteristics of the ASI system, the analysis and solution of the natural frequency ω must also use the aforementioned non-linear analysis method. The non-linear finite element method was used to analyze the structural frequency response. The nonlinear boundary element method was used to address the problem of acoustic propagation in the coupling boundary and limited acoustic field. In the coupled physical field, Ω is the closed area of a single structural field, $\Gamma_{1}$ is a type of boundary of Ω (each point has a uniquely determined tangent plane), and $\Gamma_{2}$ is another type of boundary Ω (a piecewise smooth surface with finite edges). The velocity potential function ϕ was introduced to derive the general equation for the boundary element [44,45]. The corresponding governing equations for the potential function ϕ at different positions in the coupled physical field are expressed as follows:

(11) $$\left\{\begin{array}{l} \nabla^{2}\phi =0\;,i\in \Omega \;\\ \phi =\bar{\phi }\;,i\in \Gamma_{1}\\ \partial \phi /\partial n=\bar{q}\;,i\in \Gamma_{2} \end{array}\right.$$

where $\Gamma =\Gamma_{1}\cup \Gamma_{2}$. According to the Gaussian divergence theorem and Green’s formula, the weighted residual value of the weight function $\phi^{*}$ is

(12) $$\int_{\Omega }(\nabla^{2}\phi )\phi^{*}dS=\int_{\Gamma_{2}}(\frac{\partial \phi }{\partial n}-\bar{q})\phi^{*}d\Gamma -\int_{\Gamma_{1}}(\phi -\bar{\phi })\frac{\partial \phi^{*}}{\partial n}d\Gamma$$

The boundary integral equation is established to move any point of i in the space region to the boundary region for integration. Therefore, the boundary near the point of i changes from a whole sphere to two hemispheres ($\Gamma_{\varepsilon }$ and $\Gamma^{\prime }$). The radius of the ball r=ε is very small. Equation (12) can be transformed into

(13) $$\phi_{i}=\underset{\varepsilon \to 0}{\lim}\int_{\Gamma^{\prime }}\frac{\partial \phi }{\partial n}\phi^{*}d\Gamma +\underset{\varepsilon \to 0}{\lim}\int_{\Gamma_{\varepsilon }}\frac{\partial \phi }{\partial n}\phi^{*}d\Gamma -\underset{\varepsilon \to 0}{\lim}\int_{\Gamma^{\prime }}\phi \frac{\partial \phi^{*}}{\partial n}d\Gamma -\underset{\varepsilon \to 0}{\lim}\int_{\Gamma_{\varepsilon }}\phi \frac{\partial \phi^{*}}{\partial n}d\Gamma$$

After the boundary integral equation is discretized by elements including the meshing and discretization of the integral within the domain, the matrix form of the boundary integral equation of the displacement rate is expressed as follows:

(14) $$H\dot{u}=G\dot{t}+D\dot{\varepsilon }$$

where H and G represent the kernel and interpolation shape functions of the basic solution, respectively. Matrix D is obtained from the volume fraction of the inelastic strain. $\dot{u}$, $\dot{t}$, and $\dot{\varepsilon }$ represent the displacement rate of the boundary node, surface force rate, and strain rate of the node in the domain, respectively. The stress integral equation in the domain is

(15) $$\dot{\sigma }=G^{\prime }\dot{t}-H^{\prime }\dot{u}+(D^{\prime }+C^{\prime }){\dot{\varepsilon }}^{p}$$

Substituting the boundary conditions into Equation (14), it can be rewritten as

(16) $$\dot{y}=K{\dot{\varepsilon }}^{p}+\dot{m}$$

where

(17) $$\left\{\begin{array}{l} K=A^{-1}D\\ \dot{m}=A^{-1}\dot{f} \end{array}\right.$$

Correspondingly, Equation (15) can be rewritten as:

(18) $$\dot{\sigma }=-A^{\prime }\dot{y}+{\dot{f}}^{\prime }+(D^{\prime }+C^{\prime }){\dot{\varepsilon }}^{p}$$

Substituting Equation (16) into Equation (18), we obtain:

(19) $$\dot{\sigma }=(E-A^{\prime }K){\dot{\varepsilon }}^{p}+{\dot{f}}^{\prime }-A^{\prime }\dot{m}$$

The load factors for solving the nonlinear boundary element are required to solve the load increment problem. Equations (16) and (18) can be rewritten as

(20) $$\left\{\begin{array}{l} \dot{y}=K({\dot{\varepsilon }}^{p}+\Delta {\dot{\varepsilon }}^{p})+(\lambda_{k}-\lambda_{k-1})m\\ \dot{\sigma }=-A^{\prime }\dot{y}+(\lambda_{k}-\lambda_{k-1})f^{\prime }+E({\dot{\varepsilon }}^{p}+\Delta {\dot{\varepsilon }}^{p}) \end{array}\right.$$

where ${\dot{\varepsilon }}^{p}$ represents the structural strain rate of the basic incremental step, $\Delta {\dot{\varepsilon }}^{p}$ denotes the incremental structural strain rate, and $\lambda_{k}$ and $\lambda_{k-1}$ indicate the load factors of the adjacent loading step. By setting ${\dot{\varepsilon }}^{p}+\Delta {\dot{\varepsilon }}^{p}=0$ and $\lambda_{k}-\lambda_{k-1}=1$, the boundary node stress is obtained. The load factor of the elastic loading step is then obtained by calculating the equivalent stress of the node:

(21) $$\lambda_{0}=\sigma^{y}/{\bar{\sigma }}^{\max}$$

where the maximum equivalent stress ${\bar{\sigma }}^{\max}$ is equal to the yield limit of uniaxial tension. Subsequent incremental iterative calculations were performed based on the load factor. According to the expressions for the boundary integral and displacement rates, the weak singular integral of type $1/r^{2}$ can be obtained as follows:

(22) $$I=\int_{V_{m}}\sigma_{ijk}^{s}(x)\Phi_{\alpha }(x)dV(x)$$

The second-order tightly supported radial basis function φ(r) is used for interpolation, and we obtain:

(23) $$\left\{\begin{array}{l} \tilde{I}(x)=\sum_{i=1}^{N}\alpha (x_{i})\varphi \left(r\right)\\ \varphi (r)=\max{\left\{0,(1-r)\right\}}^{4}(4r+1)\\ r=\frac{\sqrt{{\left(x-x_{i}\right)}^{2}+{\left(y-y_{i}\right)}^{2}}}{d_{mI}} \end{array}\right.$$

where $x_{i}$ is the position coordinate of the interpolation control point i, $\alpha (x_{i})$ is the expansion coefficient of the corresponding control point, and r is the support radius defined in Euclidean space. $d_{mI}$ reflects the influence range of the basis function at the control point $\left(x_{i},y_{i}\right)$. Combining Equations (22) and (23) yields

(24) $$\frac{\partial I}{\partial x_{i}}=\sum_{i=1}^{N}\frac{\partial \alpha (x)}{\partial x_{i}}\varphi (r)+\sum_{i=1}^{N}\alpha (x)\frac{\varphi (r)}{\partial x_{i}}$$

where x and r are the interpolation and displacement vectors, respectively [46,47].

## 3. Topology Optimization for Fundamental Frequency of Porous Acoustic Metamaterial Structure

The fundamental frequency is the first order (lowest) natural frequency of a porous acoustic structure. It can reflect the acoustic performance of the ASI system and is one of the key indicators for studying porous acoustic metamaterials. If the fundamental frequency can be raised higher than the external excitation frequency, the natural frequencies will deviate from the external excitation frequency and resonance phenomena will not occur. Based on practical working conditions, the fundamental frequency of the porous acoustic structure can be optimized and adjusted to avoid resonance, reduce noise, and improve the stability of the acoustic system. Therefore, the FIP design of the FMAM is performed based on the acoustic wave propagation law of the ASI system in this study. Moreover, the effects of the structural parameters and applied boundary conditions on the fundamental frequency of the new topological configuration were investigated. In the FIP design of FMAM, the first step included designing the initial structure with the largest fundamental frequency through topology optimization.

### 3.1. Modelling for Topology Optimization

**Figure 1:**
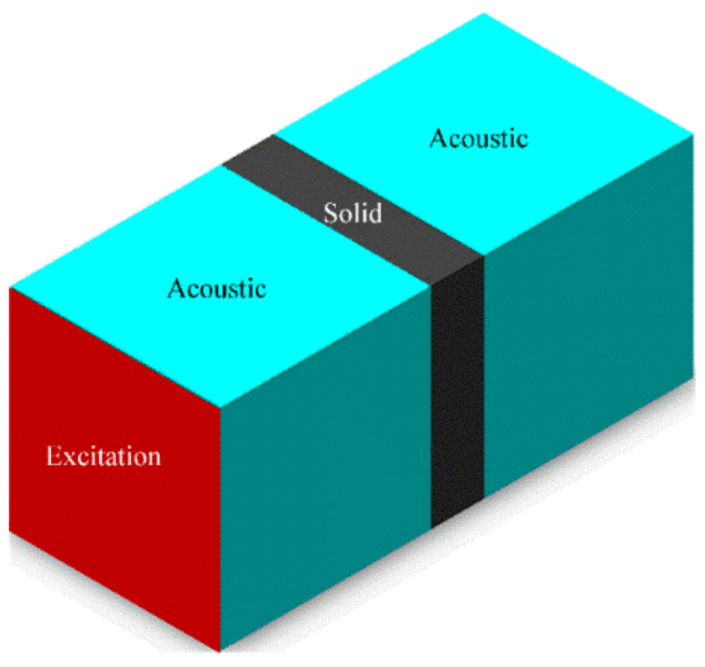
ASI system.

To facilitate the analysis, it was necessary to make the following assumptions. (a) The size of the porous acoustic structure was relatively small compared to the wavelength of the acoustic wave. (b) The integral boundary and reference point in the acoustic calculation remained unchanged during the optimization. (c) The ASI system was weakly coupled, and only considered the effect of the acoustic field on the structure, while the reaction of the structure on the acoustic field was ignored. All the simulations and calculations were executed on a computer equipped with an Intel Corei7 CPU with 2.9 GHz and 16 GB of RAM. MATLAB R2021a (The MathWorks, Inc. Location: Nedick, MA, USA) was used in the topology optimization. ANASYS R18.2 (ANSYS, Inc. Location: Pittsburgh, PA, USA) was used in the simulation. As shown in Figure 1, an ASI system is a bounded closed system. The red surface (excitation) represents the emission surface of the acoustic wave excited by the acoustic source. The two blue spaces (acoustic) represent the acoustic fields of the standard air medium. The gray (solid) area represents the FMAM design space. These components comprise the ASI system. The solid design domain of FMAM was sandwiched between the acoustic fields on either side, while the actual ASI interface lied on both sides. The surrounding areas that were not in contact with the acoustic field were fixed support areas.

The goal of topology optimization in this study was to maximize the fundamental frequency of the solid design domain in the ASI system, i.e., the fundamental frequency of the black structure in Figure 1. The optimization model constructed using the solid isotropic microstructures with penalization (SIMP) method is as follows [46,47]:

(25) $$\begin{array}{l} Find:\;\;\;\;\;\;\;\;\;\;\;\;\;\;\;\;\;\;\;\;\;\;\;\;\;\;\;\;X={\left\{x_{1},x_{2},\ldots ,x_{n}\right\}}^{T}\in \Omega \\ Max:\;\;\;\;\;\;\;\;\;\;\;\;\;\;\;\;\;\;\;\;\;\;\;\;\;\;\;\;\{\beta \}\\ Subject\;\;to:\;\;\;\;\;\;\;\;\;\;\;\;\;\;\;\;\;\;\beta -\omega_{j}^{2}\leq 0\\ \;\underset{j=1,2,\cdot \cdot \cdot ,J}{F}=(K-\omega_{j}^{2}M)u\\ \;\varphi p=Gu-Hp\\ \;\;\;\;\;\;\;\;\;\;\;\;\;\;\;\;\;\;\;\;\;\;\;\;\;\;\;\;\;\;\;\;\;\;\;\;\;\sum_{i=1}^{n}x_{i}v_{i}-fV\leq 0\;\;\;\;\;\;\;\;\;\;\;\;\;\;\;\;\;\;\;\;\;\;\;\;\;\;\;\;\;\;\;\;\;\;\;\;\;\;\;\;\\ \;\;\;\;\;\;\;\;\;\;\;\;\;\;\;\;\;\;\;\;\;\;\;\;\;\;\;\;\;\;\;\;\;\;\;\;\;0<x_{min}\leq x_{i}\leq 1\;\;\;,\;\;\;\;(i=1,2,3,\ldots ,n) \end{array}$$

where β represents the objective function that is a scalar factor, X denotes the matrix of free variables, $x_{i}$ represents the relative density of the material element, $v_{i}$ indicates the relative volume of the material element, V refers to the overall structural volume, and f is the constraint of the material volume fraction. $\omega_{j}$ is the natural frequency of the *j*th order, and $\omega_{j}^{2}$ represents the eigenvalue. The scalar factor β is introduced as an auxiliary design variable and objective function in conjunction with the first formula in the constraint condition. To achieve the design goal of this study, the maximization of the fundamental frequency was expressed through the maximization of β. Here, a sensitivity filter scheme is used to avoid the dependency on mesh size and initial design.

When performing a sensitivity analysis for the above optimization model, the first two equations of constraint conditions in Equation (25) were derived using the design variables, and Equation (26) was obtained:

(26) $$\left\{\begin{array}{l} F^{\prime }=(K-\omega_{j}^{2}M{)}^{\prime }u+(K-\omega_{j}^{2}M)u^{\prime }\\ \varphi^{\prime }p+\varphi p^{\prime }=G^{\prime }u+Gu^{\prime }-H^{\prime }p-Hp^{\prime } \end{array}\right.$$

The integral boundary and reference points in the calculation process remained unchanged. Moreover, the external excitation was an independent load (the load vector belongs to the external input condition, which is independent of the design variable). $F^{\prime }$, $G^{\prime }$, and $H^{\prime }$ were all equal to 0. A sensitivity analysis of the natural frequency was obtained by deriving the second equation of the constraint condition in Equation (26). The sensitivity of the objective function $\omega_{1}$ relative to the design variable $x_{i}$ is as follows [48]:

(27) $$\frac{\partial \omega_{j}}{\partial x_{i}}=(\frac{K}{2\omega_{j}M}-\frac{\omega_{j}}{2})\frac{\partial u}{\partial x_{i}}-\frac{\omega_{j}}{2M}\frac{\partial M}{\partial x_{i}}+\frac{1}{2\omega_{j}M}\frac{\partial K}{\partial x_{i}}$$

The optimality criterion (OC) algorithm was used to solve the above optimization model after obtaining the sensitivity result shown in Equation (27). Non-linear finite element and boundary element analyses were performed for the aforementioned optimization model. The hybrid iterative method was used in the non-linear finite element:

(28) $$\left\{\begin{array}{l} K(u)\Delta u=\Delta F\\ K{\ddot{u}}^{n+1}=F^{n+1}-Ku^{n+1} \end{array}\right.$$

The nonlinear boundary element iteration equation of ASI interface is as follows:

(29) $$\left\{\begin{array}{l} \dot{y}=K({\dot{\varepsilon }}^{p}+\Delta {\dot{\varepsilon }}^{p})+(\lambda_{k}-\lambda_{k-1})M\\ \dot{\sigma }=-A^{\prime }K{\dot{\varepsilon }}^{p}+\dot{M}+(\lambda_{k}-\lambda_{k-1})F^{\prime }+E({\dot{\varepsilon }}^{p}+\Delta {\dot{\varepsilon }}^{p}) \end{array}\right.$$

### 3.2. Topology Optimization

**Figure 2:**
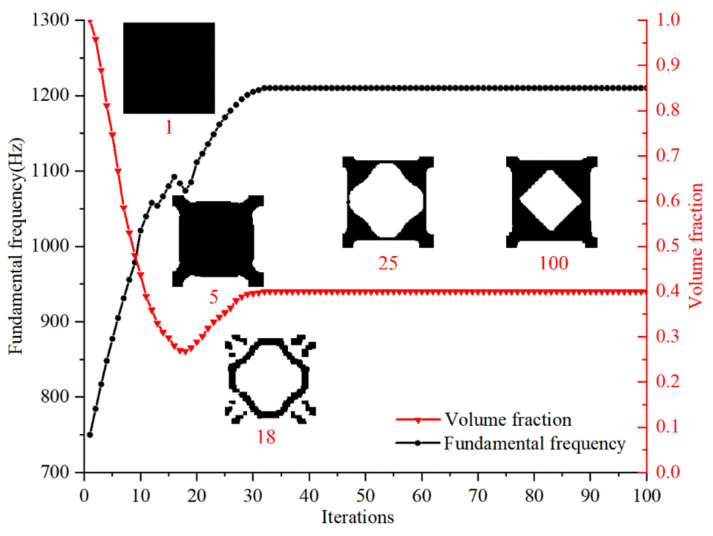
Iterative process of topology optimization, which also shows the structural topologies of the 1st iteration, 5th iteration, 18th iteration, 25th iteration and 100th iteration.

The optimization model is presented in Equation (25). An acoustic wave with a frequency of 500 rad/s was used as the effective excitation of the design domain. The volume constraint of the solid design domain was set to 0.4. The iterative process of the objective function, volume fraction of the design domain, and topology configuration in the topology optimization of FMAM are shown in Figure 2. As shown in Figure 2, the initial design (denoted by “1”) is a completely solid square meshed by 100 × 100 grids. Here, we also used different initial designs and different meshes to implement the topology optimization. It is found that to the initial configurations and the mesh size has a very limited impact on the optimized topology of FMAM, due to the use of a sensitivity filter.

It can be observed that the fundamental frequency of FMAM gradually increases while the volume fraction gradually decreases as the topology optimization continues. The relevant parameters (objective function and volume fraction) gradually converged, and the topological configuration gradually stabilized when the optimization model was iterated to 35 steps. The average optimization time for single cell is 217 s. Moreover, the fundamental frequency of the new configuration of the FMAM obtained after topology optimization exhibited a comparatively large increase (from 750 to 1210 Hz). This result indicated that the structural fundamental frequency of porous acoustic materials could be effectively adjusted through topology optimization, and a new FMAM configuration could be obtained. However, the range of mid-low-frequency noise that is ubiquitous in actual production and life is 500–2000 Hz. The results of topology optimization cannot fully cover this range. Therefore, to further improve the fundamental frequency of the FMAM, it was necessary to further optimize its parameters, as discussed in the following section.

**Figure 3:**
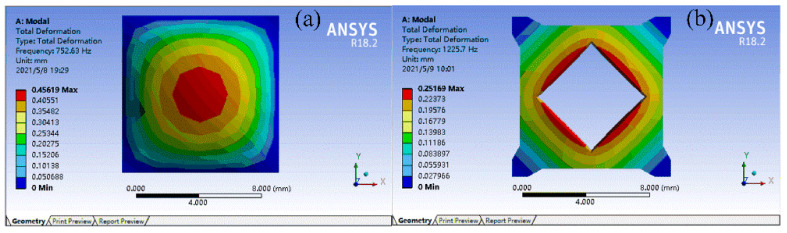
The nephograms of modal simulation for unit cell of FMAM: (**a**) represents the initial structure; (**b**) represents the optimal structure.

To verify the effect of the above topology optimization for FMAM in improving the fundamental frequency, a modal analysis of the new configuration obtained by topology optimization was performed. The nephograms of the modal simulation for the unit cell of the FMAM are shown in Figure 3, where (a) represents the initial structure and (b) represents the optimal structure. It can be observed that the fundamental frequencies of the initial and optimal structure are 752.63 and 1225.7 Hz, respectively, which is basically the same as the fundamental frequency change law shown in Figure 2. This also indicates that the fundamental frequency maximization model of the FMAM established in this study is correct.

**Figure 4:**
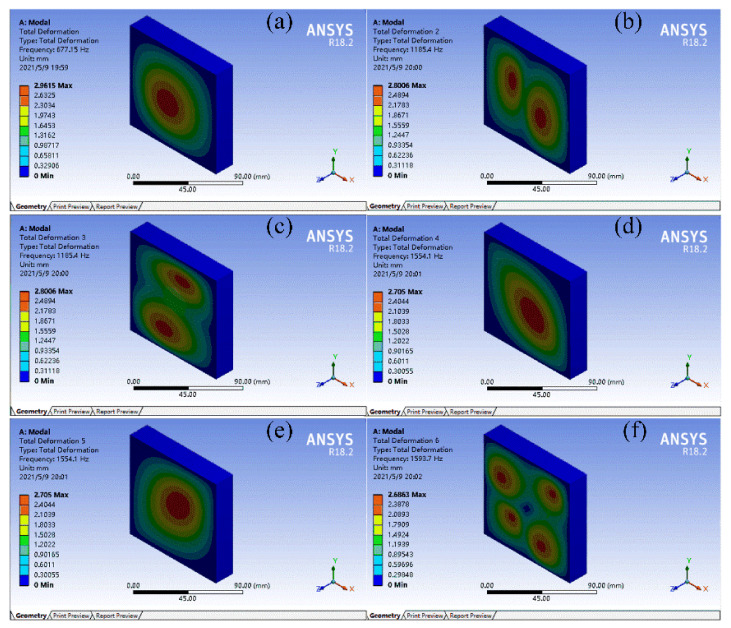
Nephograms of modal simulation for 10 × 10 multi-cell of FMAM before topology optimization: (**a**–**f**) represent the first six modes of the FMAM in the ASI system respectively.

**Figure 5:**
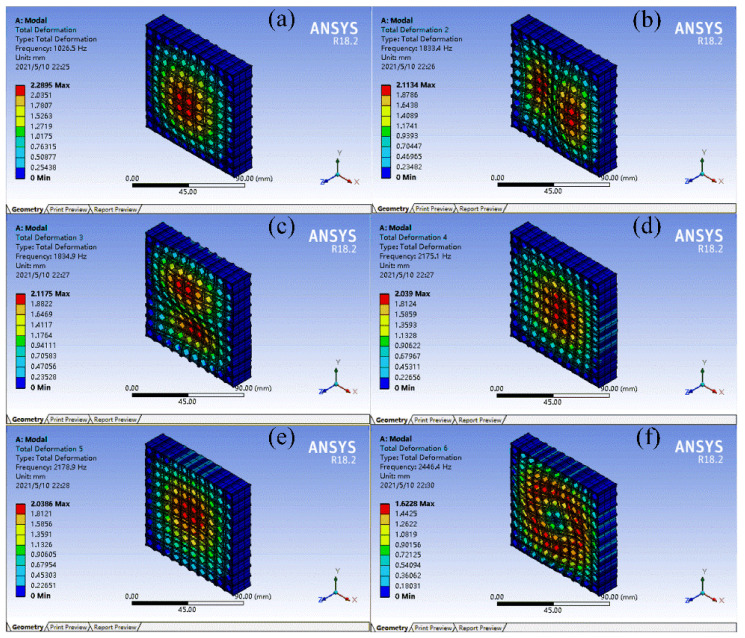
Nephograms of modal simulation for 10 × 10 multi-cell of FMAM after topology optimization: (**a**–**f**) represent the first six modes of the FMAM in the ASI system, respectively.

FMAMs generally have a multicell structure for practical applications. To verify the effect of the topology-optimized FMAM in the actual application process, a 10 × 10 multicell structure was constructed for modal simulation. In the modal analysis, low-order modes had a greater impact on the vibration system. Therefore, the first six modes of the FMAM in the ASI system were analyzed. The nephograms of the modal simulation for 10 × 10 multi-cells of the FMAM are shown in Figure 4 and Figure 5, where Figure 4 represents the initial structure while Figure 5 represents the optimal structure. Compared to Figure 3, we find that the fundamental frequency of the initial (677.15 Hz) and optimal (1026.5 Hz) structures of the 10 × 10-FMAM were reduced. This was because the fundamental frequency of the structure was related to its hardness, quality, and dimensions.

The modal analysis results of the FMAM are presented in Table 1. By analyzing the relevant data in Figure 4 and Figure 5 and Table 1, we observed that the FMAM increased its natural frequency and reduced its maximum amplitude through topology optimization. These results demonstrated that the topology optimization for the fundamental frequencies of the porous acoustic metamaterials investigated in this study could effectively increase their natural frequencies and obtain FMAM with excellent modal characteristics. However, the former three natural frequencies of FMAM (1026.5 Hz, 1833.4 Hz, and 1834.9 Hz, respectively) remained unable to avoid the mid-low-frequency noise distribution range [500 Hz, 2000 Hz]. Therefore, it was necessary to further improve the fundamental frequency of the FMAM using the following parametric optimization:

## 4. Parametric Optimization Based on Surrogate Model

### 4.1. The Theoretical Background of Surrogate Model

The SM used in this study was based on the effective global optimization (EGO) of the Kriging model. The Kriging model is an unbiased estimation model that integrates the regression model and enables a random process to predict the values of minimum variance and sample points. Consequently, an SM can depict the approximate original model smoothly with only a few sample points and is suitable for highly non-linear issues [49,50,51]. In the Kriging model, the true relationship between the response values ($Y={\left[y_{1},y_{2},\cdot \cdot \cdot ,y_{m}\right]}^{T},(y_{i}\in R^{q})$) and independent variables ($X={\left[x_{1},x_{2},\cdot \cdot \cdot ,x_{m}\right]}^{T},(x_{i}\in R^{n})$) of a system can be expressed as follows:

(30) Y(X)=g(X)+Z(X)

where $g(X){=[g}_{1}(X{),g}_{2}(X),\cdot \cdot \cdot {,g}_{k}(X{)]}^{T}$ is a deterministic part that can be expressed as k, a known regression function. Z(X), called as the system deviation, is independently and identically distributed (for example, it conforms to a normal distribution and has the following statistical characteristics: E[Z(X)]=0, $Var[Z(X)]=\sigma^{2}$, and $\mathrm{cov}[Z(x_{i}),Z(x_{j})]=\sigma^{2}R(c,x_{i},x_{j})$). Here, $x_{i}$ and $x_{j}$ are two arbitrary sample points, $R(c,x_{i},x_{j})$ is a spatial correlation function with parameter c, and $\sigma^{2}$ is the process variance. The common related functions are Gaussian functions, $R(X)=\exp(-X^{2}/c^{2})$, R(X)=exp(−X/c), and so on. c can be solved using the following equation:

(31) $$\min\{\varphi (c)=-(m\ln\sigma^{2}+\ln\left|R\right|)/2\}$$

where R is a correlation matrix. Linear weighted superposition interpolation of the response value $y_{i}$ of sample point $x_{i}$ is applied to calculate the response value of the measuring point (X):

(32) $$\hat{Y}(X)=w{\left(X\right)}^{T}Y$$

where $w(X)=(w_{1},w_{2},\cdot \cdot \cdot ,w_{n})$ denotes the weight coefficient of the solution. By integrating the Lagrange multiplier method in accordance with the unbiased and minimum variance conditions, the final weight coefficient vector is

(33) $$w(X)=R^{-1}(r(X)+G{\left(G^{T}R^{-1}G\right)}^{-1})\cdot (G^{T}R^{-1}r(X)-g(X))$$

where $G={\left[g^{T}(x_{1}),g^{T}(x_{2}),\cdot \cdot \cdot ,g^{T}(x_{n})\right]}^{T}$ is an n × k expansion matrix and $g^{T}(x_{i})$ is the transpose of g(X). Integration of Equation (33) into Equation (30) results in the following expression for the Kriging model:

(34) $$\hat{Y}(X)=g(X)\beta^{*}+r{\left(X\right)}^{T}R^{-1}(Y-G\beta^{*})$$

where $\beta^{*}={\left(G^{T}R^{-1}G\right)}^{-1}G^{T}R^{-1}Y$ is the least-square estimation of β,$R={\left[R_{ij}\right]}_{n\times n}={\left[R(c,x_{i},x_{j})\right]}_{n\times n}$, and $r(X)={\left(R(c,x_{1},X),R(c,x_{2},X),\cdot \cdot \cdot ,R(c,x_{n},X)\right)}^{T}$.

Based on the sequence optimization of Kriging-SM, the SM was revised until it converged by means of continuous optimization of the design and addition of sample sets. The sampling criterion was directly related to the speed of convergence and stability, which are essential for optimization. At present, the common sampling criteria are mainly based on a multi-start search with geometric global exploration (MSG) and uncertainty-based EGO [52] that is more popular and applied here. The EGO algorithm is based on Kriging-RSM and the expected improvement (EI) function, wherein the EI criterion is utilized to guide the updating and optimization of the kriging model. An optimal response value based on the current design can be obtained, and points with larger prediction errors can be obtained by maximizing its expectation.

### 4.2. The Process of Parametric Optimization

In the process of parametric optimization based on SM, the initial configuration of the FMAM obtained by topology optimization was first reconstructed in SolidWorks to express the typical shape and size of the material characteristics. Moreover, ANSYS Workbench and COMSOL were used to perform the optimization analysis of the specific target performance on the new model (such as FMAM, which requires modal analysis, especially structural fundamental frequency analysis) and record it in the transfer file. Finally, the corresponding information files were transferred into the SM constructed using MATLAB for optimization analysis and repeated corrections. The entire parametric optimization process of the FMAM was expected to be completed before the overall optimization result reached the feasible region and the optimization program converged.

**Figure 6:**
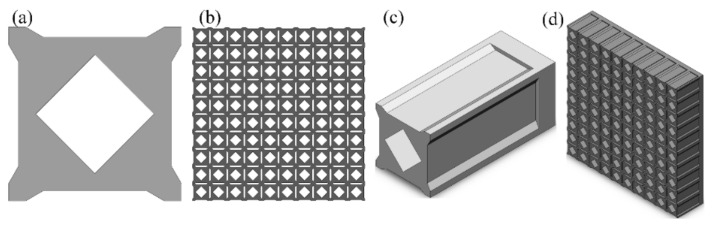
Composition of three-dimensional honeycomb porous FMAM: (**a**) the remodeled two-dimensional configuration obtained through the topology optimization; (**b**) the cross-sectional shape of the three-dimensional cellular porous FMAM; (**c**) the three-dimensional unit cell; (**d**) the 10 × 10 array three-dimensional honeycomb porous structure.

Parametric modeling is the first step in parametric optimization. The aforementioned optimization model was reconstructed based on the honeycomb porous structure. The typical shape and size parameters of the reconstructed structures were extracted to enhance the adjustment effect of the fundamental frequency and improve the dynamic characteristics of the FMAM through parametric optimization. Therefore, the two-dimensional configuration obtained through the topology optimization above was remodeled first, as shown in Figure 6a. Subsequently, the three-dimensional unit cell was stretched in the third dimension during its practical application, as shown in Figure 6c, based on the cross-sectional shape in Figure 6a. Finally, the three-dimensional unit cell in Figure 6c was arranged in a 10 × 10 array to form the three-dimensional honeycomb porous structure shown in Figure 6d. Figure 6b shows the cross-sectional shape of the three-dimensional cellular porous FMAM.

**Figure 7:**
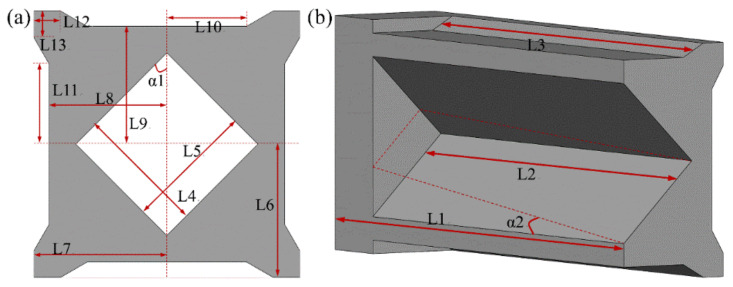
Parametric model of three-dimensional unit cell of FMAM: (**a**) the cross-section of the three-dimensional honeycomb porous; (**b**) the internal section of the three-dimensional honeycomb porous.

The honeycomb porous structure was constructed using a large number of repeated unit cells, which are the basic function and size unit. Therefore, the parametric definition of the structural unit cell reflected the size information of the entire three-dimensional honeycomb porous FMAM. The cross-section of the three-dimensional honeycomb porous FMAM investigated in this study was also a symmetrical structure. Therefore, a quarter of the structure was used to define the relevant parameters, as shown in Figure 7. In Figure 7, the section shape of the unit cell defines 11 independent parameters (L4–L13 and α1), and the depth of the cell defines four independent parameters (L1–L3 and α2). A total of 15 independent parameters were used for parametric optimization in this study. The various physical parameters of the ASI system are listed in Table 2. Table 3 lists the values of the 15 main structural parameters.

The design goal of parametric optimization based on the SM was to maximize the fundamental frequency of the FMAM. According to Figure 6, there are 15 independent parameters (L1–L13, α1–α2) extracted by the parameterization of FMAM. Different structural parameters were combined into the initial sample points, which were then used as the input variables for the SM. The fundamental frequency $\omega_{n}$ was the output variable corresponding to the sample point and was used to evaluate the performance of the FMAM to avoid resonance and maintain the stability of the structure. Structures with 10 × 10 arrays were used in the SM simulation process. These structures were more in line with the actual application situation, and the force status included the interaction forces between the unit cells.

In addition, the fundamental frequency could not be raised at the expense of the overall effect of noise reduction of the porous acoustic metamaterial in the process of FMAM optimization. Therefore, it was necessary to add sound pressure level (SPL) to the exit surface of the FMAM as a constraint condition in the optimization. However, if this constraint was added to the topology optimization model of FMAM or a multi-objective topology optimization model was established, it would significantly increase modeling difficulties, reduce optimization efficiency, and make the optimization results difficult to converge. Therefore, in this section, the SPL on the exit surface of the FMAM was added as a constraint to the parametric optimization. SM was utilized to solve this problem, effectively improving the design efficiency. Based on the above requirements, the following optimization model was established:

(35) $$\begin{array}{l} \max\;\;\;\;\;\;\;\;\;\;\;\;\omega_{n}(x)\;\;\;\;\\ s.t.\;\;\;\;\;\;\;\;\;\;\;\;SPL_{out}(x)<f_{coeff}\cdot SPL_{ini}\\ \;\;\;\;\;\;\;\;\;\;\;\;\;\;\;\;\;\;\;\;\;x=\{x_{i}\}\\ \;\;\;\;\;\;\;\;\;\;\;\;\;\;\;\;\;\;\;\;\;\;\;x_{iL}\leq x_{i}\leq x_{iU},\;i=1,2,\cdots ,N\\ \;\;\;\;\;\;\;\;\;\;\;\;\;\;\;\;\;\;\;\;\;\;\;\omega_{1}\geq \omega_{ass}\;\;\;\;or\;\;\;\;\Delta \omega_{1}/\omega_{ass}\leq 0.001\\ \;\;\;\;\;\;\;\;\;\;\;\;\;\;\;\;\;\;\;\;\;\;\;n=1,2,\cdots ,6 \end{array}$$

where $\omega_{n}$ represents the natural structural frequency, $\omega_{1}$ represents the fundamental structural frequency, and $\Delta \omega_{1}$ is the difference of $\omega_{1}$ between two successive iterations. $\omega_{ass}$ is the specified fundamental frequency adjustment interval, which was set to [500 Hz, 2000 Hz] in this study. $x=\{x_{i}\}$ indicates the structural variables and $x_{iL}$ and $x_{iU}$ are the upper and lower limits of the ith structural variable, respectively. $SPL_{out}(x)$ represents the SPL on the exit surface of an optimized structure during the parametric optimization while $SPL_{ini}$ represents the SPL on the exit surface of an initial structure during the parametric optimization process. $f_{coeff}$ is a customized acoustic wave reduction coefficient generally set in the range of [0.7, 0.9]. To avoid excessive pressure on the constraint conditions in the optimization process and ensure the optimization effect, $f_{coeff}=0.9$ was used in this study.

A Latin hypercube design was used to obtain an efficient and reasonable sample space in the SM. The factor-level distribution of each sample point in the matrix was as uniform as possible to guarantee the accuracy of the SM in the entire design space. Upon comparing all feasible algorithms, relevant scholars agreed that if the sample space of the SM started with a 5-dimensional function evaluation and completed with an 11-dimensional accurate function evaluation, it could converge to obtain an optimization result that meets the accuracy requirements. In this study, D = 15. The initial sample space required 75 points, and there was a total of 165 points after the optimization. The SM still required to calculate 90 points to complete the parametric optimization.

### 4.3. Results Analysis of Parametric Optimization

**Figure 8:**
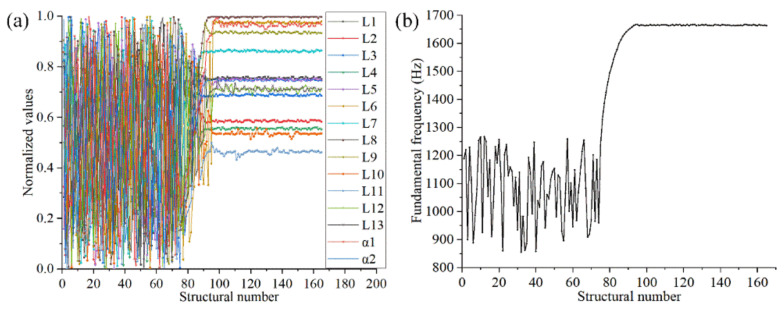
Change trend of input (structural) and output parameters in SM: (**a**) the different parameters fluctuate in different states during the convergence process; (**b**) the fundamental frequency of the FMAM.

After the optimization of the FMAM based on the SM, the optimal values of the main design parameters listed in Table 4 were obtained. The structural parameters of FMAM exhibited different value ranges. To compare and observe the changing process of various structural parameters at the same scale, we normalized the structural parameters during the optimization process. The changing trends of the main design parameters in the optimization process of the SM are shown in Figure 8.

As shown in Figure 8a, different parameters fluctuate in different states during the convergence process, and their sensitivities to the changes in the optimization model are different. A higher fluctuation of the design variables led to a stronger independence and lower sensitivity to changes in the SM. Therefore, the parameters with large fluctuations could be excluded when adjusting the SM for an extensive parametric optimization. As shown in Figure 8b, the fundamental frequency of the FMAM approaches a stable value of 1665 Hz after FMAM is optimized by the SM. Observing the fundamental frequency of 10 × 10-FMAM (1026.5 Hz) and parameter combinations in the initial sample space [854.5 Hz, 1266.9 Hz]. It is indicated that the fundamental frequency of FMAM optimized by SM has a larger amplitude increase. It is possible to deviate from the mid- to low-frequency noise distribution range [500 Hz, 2000 Hz] and avoid structural resonance hazards in daily life. In addition, it could be explained that the parametric optimization based on the SM in this section reached the structural optimization goal of FMAM.

**Figure 9:**
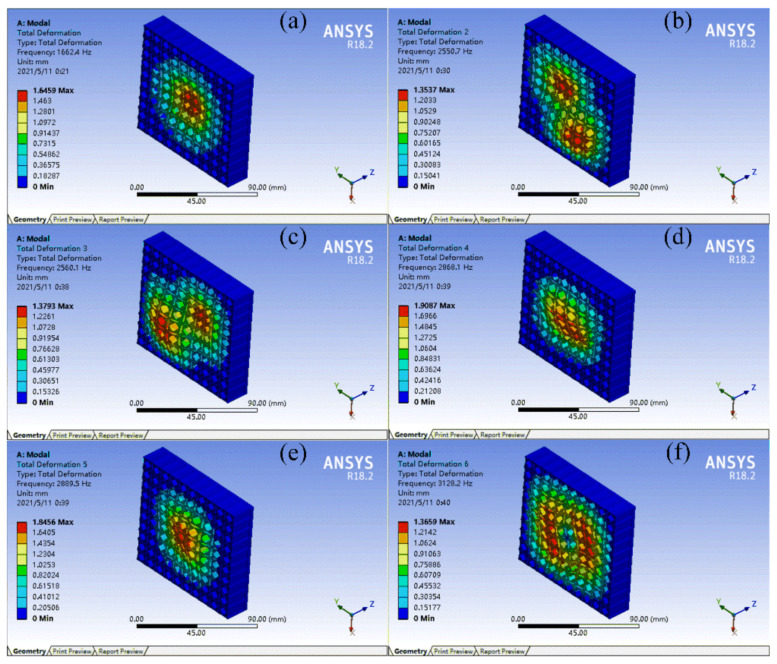
Nephograms of modal simulation for 10 × 10 multi-cell of FMAM after parametric optimization: (**a**–**f**) represent the first six modes of the FMAM in the ASI system, respectively.

Figure 9 shows the nephograms of the modal simulation for 10 × 10 multicells of the FMAM after parametric optimization. Table 5 shows the results of the six modal analyses for the porous acoustic metamaterials before and after parametric optimization. First, we found that the FMAM further improved its natural frequency and reduced its maximum amplitude through parametric optimization by analyzing the relevant data in Figure 9 and Table 5. The structural fundamental frequency increased the most (61.95%) compared with the others. Next, the fundamental frequency of FMAM after parametric optimization was 1662.4 Hz, consistent with the structural fundamental frequency (1665 Hz) optimized by the SM. Lastly, the fundamental frequency (1662.4 Hz) of the FMAM optimized by parametric optimization remained in the mid-to low-frequency noise distribution range [500 Hz, 2000 Hz], while the other higher-order natural frequencies crossed this range. Simultaneously, the actual frequency associated with the fundamental frequency of the FMAM decreased to [1550 Hz, 2000 Hz], capable of meeting most noise application environments. Thus, the above conclusions indicated that the SM established in this section could increase the natural frequency of the FMAM and effectively avoid structural resonance. However, the FMAM still needs to be further optimized and improved for a more demanding noise-working environment.

**Figure 10:**
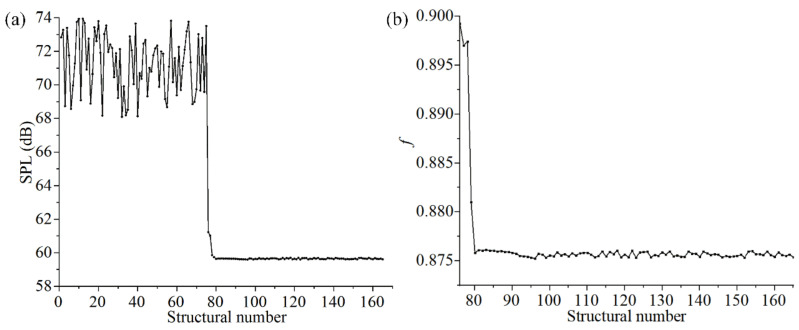
Change trend of SPL and f during the parametric optimization: (**a**) represents SPL; (**b**) represents f.

Moreover, the fundamental frequency could not be increased at the expense of the overall effect of noise reduction for porous acoustic metamaterials in FMAM optimization. Therefore, the SPL on the exit surface of the FMAM was added as a constraint condition in the optimization to tune the overall vibration and noise reduction effect of the porous acoustic metamaterial. To explain the change in the SPL on the exit surface during the parametric optimization of the FMAM, we defined the actual reduction coefficient $f=\frac{SPL_{out}(x)}{SPL_{ini}}$ of SPL. Figure 10 shows the changes in SPL and f during parametric optimization. It can be observed from Figure 10 that the optimization results satisfy the constraints, which are reasonable and effective during the optimization process.

### 4.4. Secondary Optimization

The FMAM obtained through the above parametric optimization did not meet the requirements of completely avoiding the mid- to low-frequency noise distribution range [500 Hz, 2000 Hz]. It was necessary to further improve the fundamental frequency of FMAM through secondary optimization. Secondary optimization removed some structural parameters with poor correlation and low sensitivity based on the relevant conclusions of the first parametric optimization. It also reduced the dimensionality of the sample points. Therefore, the newly built SM released some degrees of freedom and motivated extensive optimization goals. 

**Figure 11:**
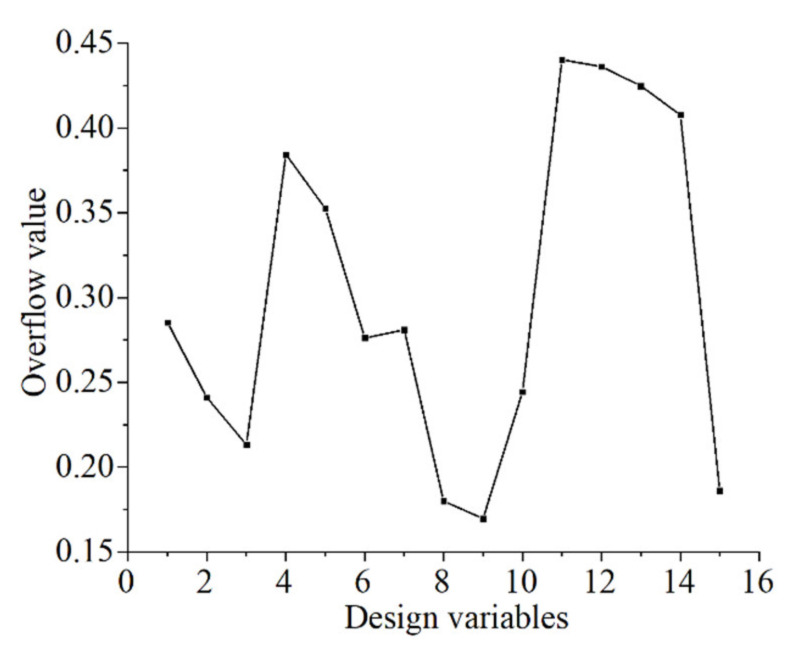
Influence of the design variables on optimization goals.

Figure 11 shows the influence curves of the design variables (L1–L13 and α1–α2) on the optimization goal during the initial parametric optimization. The overflow value (the sum of the absolute difference between the actual and average values) represents the influence of the design variable on the SM. A larger overflow value leads to a smaller impact. Design variables with large overflow values must be eliminated first when the optimization model is subsequently improved. The critical overflow value in this study was 0.3. Six parameters (L4–L5, L11–L13, and α1) exceed the critical overflow value. Therefore, the remaining nine parameters (L1–L3, L6–L10, and α2) were used as basic variables to construct the SM for the secondary optimization.

Table 6 lists the value ranges of the nine optimization parameters in the secondary optimization. Since the degrees of freedom were released by removing some parameters, the structural performance was investigated extensively on the basis of the initial optimization. The optimization results were only fine-tuned based on the first optimization results rather than deviating from the overall direction of the first optimization. Therefore, the original optimized value was centered, and 10% of the original space amplitude was floated up and down to form a new parameter value range, as shown in Table 6.

**Figure 12:**
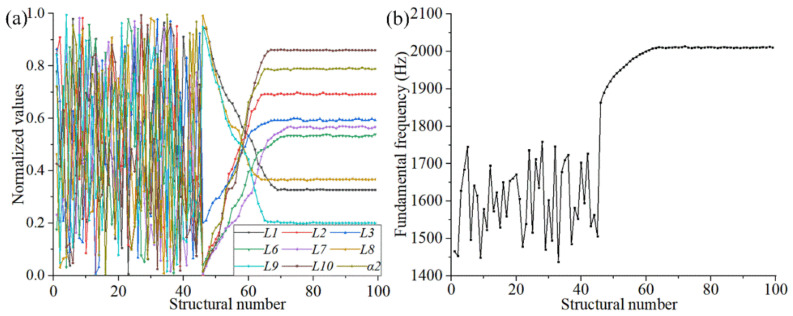
Change trend of input (structural) and output parameters in the secondary optimization: (**a**) the different parameters fluctuate in different states during the convergence process; (**b**) the fundamental frequency of the FMAM.

Figure 12 shows the changing trend of the input (structural) and output parameters in the secondary optimization. As shown in Figure 12, the structural parameters gradually converge to a new stable state after iterative updating and form a new combination of size parameters through secondary optimization of the FMAM. In addition, the structural fundamental frequency improved significantly from 1665 Hz to 2011 Hz as the output variable and optimization target. This made the entire modal frequency distribution range avoid the mid-to low-frequency noise distribution range [500 Hz, 2000 Hz]. The ASI system did not exhibit resonance in the actual application process, improving the vibration and noise reduction performance of FMAM.

## 5. Experimental Analysis of Frequency Response Based on Closed Acoustic Box

### 5.1. Prototype Manufacturing and Scheme Design

**Figure 13:**
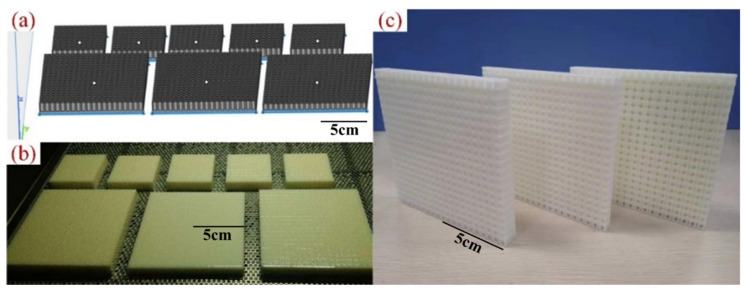
Additive manufacturing of FMAM specimens: (**a**) is the basic preprocessing model; (**b**) is the specimen manufacturing and post-processing model; (**c**) is the partial physical diagram of FMAM.

Owing to the small size and complex configuration of the porous acoustic metamaterials obtained through the above optimization, this study chose additive manufacturing (3D printing) to make FMAM. The matrix material of FMAM is a C-UV 9400E polymer. However, the number of sample points in the optimization of SM is very large, and the cost of 3D printing is high. To improve experimental efficiency, some typical structures were utilized in additive manufacturing. Figure 13 shows the AM process of additive manufacturing for FMAM specimens, where (a) is the basic preprocessing model, (b) is the specimen manufacturing and post-processing model, and (c) is the partial physical diagram of FMAM. The structures in Figure 13a are modeled by 1:10 comparing with the real structure.

**Figure 14:**
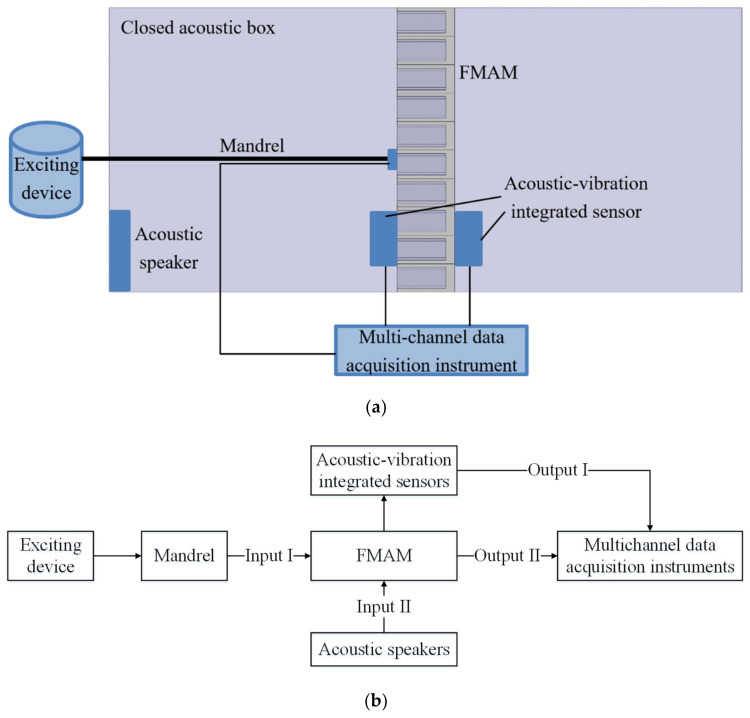
Frequency response experiments based on the closed acoustic box: (**a**) Physical layout of the frequency response experiment; (**b**) Flowchart of the frequency response experiment.

**Figure 15:**
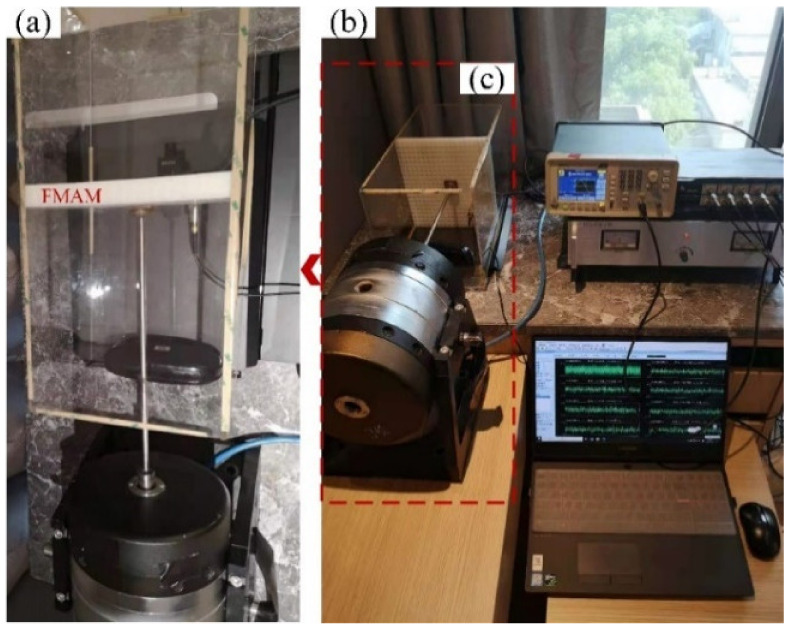
Site of frequency response experiments: (**a**) is a magnified view of (**c**); (**b**) is the complete layout site of the experimental device, (**c**) is the closed ASI interaction space connected to the vibrating device.

**Figure 16:**
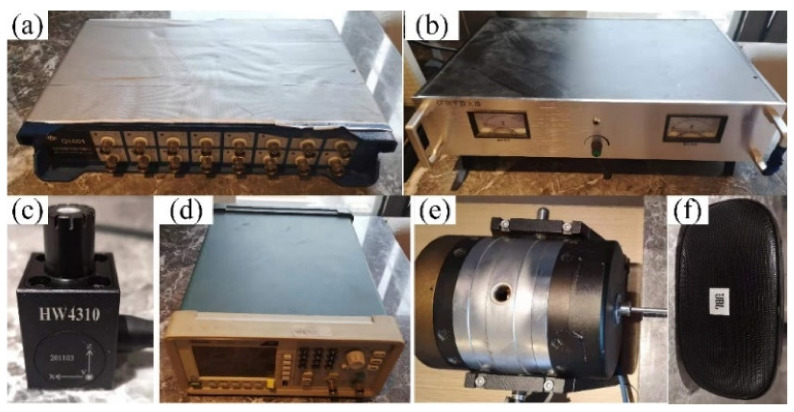
Main equipment for frequency response experiments: (**a**–**f**) represent the multichannel data acquisition instruments, power amplifiers, acoustic-vibration integrated sensors, signal generators, vibration exciters, and acoustic speakers, respectively.

To analyze the frequency characteristics and related acoustic functions of the FMAM in the ASI system, a schematic of the frequency response experiment based on a closed acoustic box was constructed, as shown in Figure 14. Figure 14a is the physical layout of the frequency response experiment. Figure 14b is the flowchart of the frequency response experiment. In this study, an external enclosed space was constructed using acrylic panels of 5 mm thickness. The relative positions of the closed acoustic box were set up with reference to Figure 1. Figure 15 shows the site of the frequency response experiments based on the closed acoustic box, where (b) is the complete layout site of the experimental device, (c) is the closed ASI interaction space connected to the vibrating device, and (a) is a magnified view of (c). The main equipment used in the frequency response experiments based on the closed acoustic box is shown in Figure 16. There are multichannel data acquisition instruments, power amplifiers, acoustic-vibration integrated sensors, signal generators, vibration exciters, and acoustic speakers represented by (a) to (f), respectively. An acoustic-vibration integrated sensor was used to monitor the noise in the enclosed space at the experimental site.

**Figure 17:**
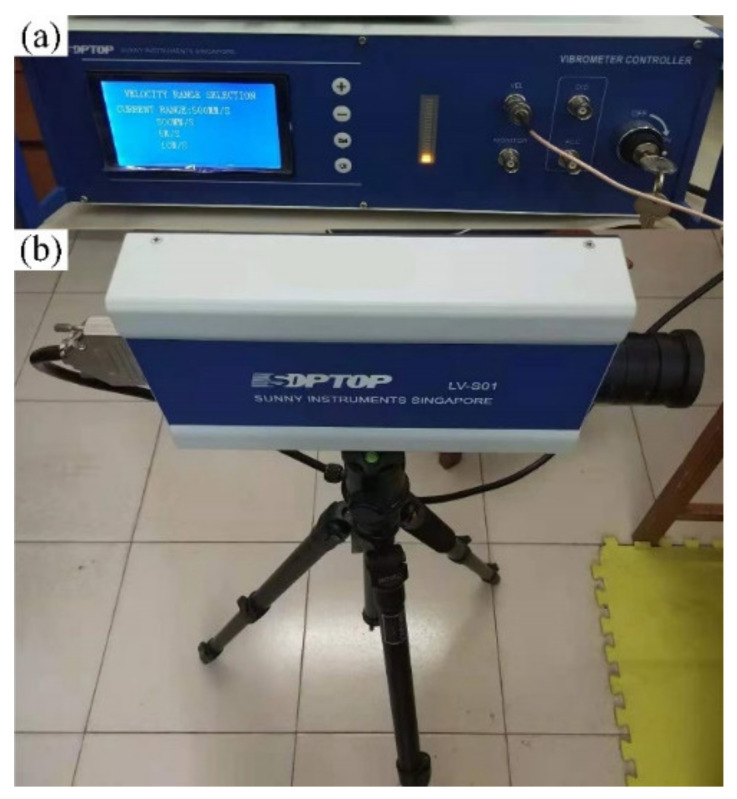
Amplitude experimental device for FMAM: (**a**) is a vibration controller used to adjust the intensity of the collected signal and (**b**) is an interferometer used to emit polarized light to measure the structural vibration.

In this study, an acoustic speaker was used as the acoustic source to output external excitation acoustic waves, equivalent to a point source with a larger area. However, the output frequency could not be directly adjusted through the control button since the acoustic speaker could only adjust the volume. Therefore, it was necessary to use an excitation device to calibrate the acoustic waves generated by the acoustic box. The control device of the acoustic box corresponded directly to the frequency of the output acoustic wave in this manner. The distribution range of the external excitation noise set in this study was [500 Hz, 2000 Hz], the output frequency range of the excitation device was [5 Hz, 3000 Hz], and the frequency range of the acoustic wave output by the speaker was [100 Hz, 8000 Hz]. To understand the adjusting effect of the resonance frequency band, it was necessary to analyze the exact position of the structural resonance frequency before and after optimization. As shown in Figure 17, this study uses the SOPTOP LV-S01 laser Doppler vibrometer to monitor the vibration amplitude of the FMAM during the excited vibration process and analyzes the resonance frequency band of the FMAM with non-contact dynamic interferometry.

### 5.2. Modal Experiment

The experimental analysis of the FMAM in the optimization process included two main aspects. The first was a comparative analysis of the experimental and simulation results. The correctness of the results in the parametric optimization was verified through the experimental results, and the process of parametric optimization was also adjusted through deviation feedback. The second was to analyze the frequency characteristics of the FMAM through related experiments, including the distribution range of the natural frequency, the determined position of the resonance frequency, and the fundamental frequency. The output vibration at the end of the ejector rod and vibration and SPL were collected on the incident and emission surfaces of the FMAM, respectively. The Acoustic testing software is Sound Express V1.0 (Beijing Hua Qing Si Chuang Acoustic Technology Co., Ltd. Location: Beijing, China).

**Figure 18:**
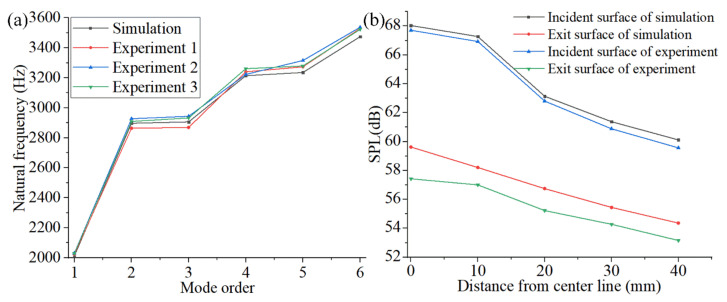
Comparison of experiment and simulation results of FMAM: (**a**) represents the natural frequency; (**b**) represents the surficial SPL.

Figure 18 shows the comparison diagrams of the experimental and simulation results for the FMAM, where (a) represents the natural frequency and (b) represents the surficial SPL. It can be observed in Figure 18a that the results of the modal experiments and simulations of the FMAM are consistent in terms of the changing trends and values. Further, it can be observed in Figure 18b that the experimental and simulation results of the FMAM are also consistent with respect to the changing trend for the SPL at different positions on the entrance and exit surfaces. The specific values also exhibited stable deviations (systematic errors).

**Figure 19:**
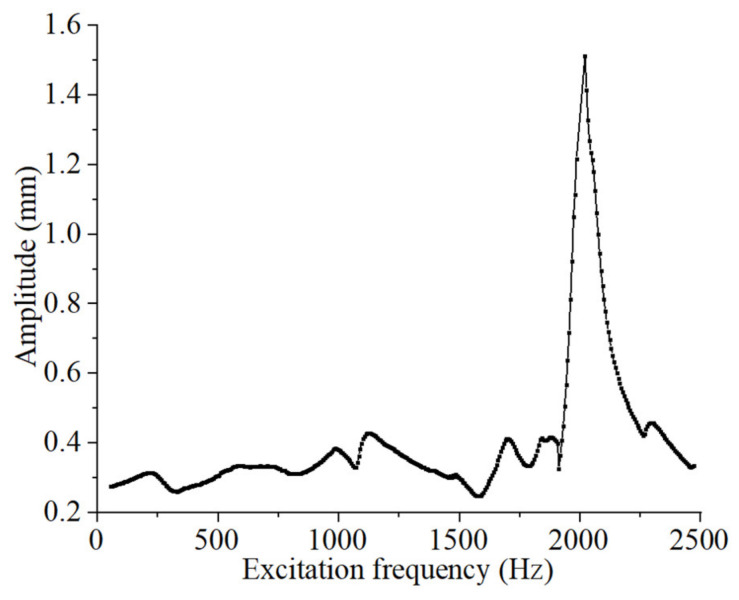
Amplitude of the incident surface corresponding to the noise impact frequency band.

Figure 19 shows the amplitude of the incident surface corresponding to the optimized FMAM in the noise impact frequency band [50 Hz, 2500 Hz]. Herein, the FMAM reaches a resonance state at 2010 Hz and the corresponding amplitude is 1.513 mm. The experimental results were consistent with the simulation results. The comparative analysis results of the above experiments and simulations demonstrated that the FIP design for the fundamental frequency of the porous acoustic metamaterial structure in this study was effective. The obtained FMAM also exhibited a good vibration and noise reduction performance.

## 6. Conclusions

To prevent the porous acoustic structures in the ASI system from resonating and affecting its vibration and noise reduction performance, this study executed an FIP design of the structural fundamental frequency of porous acoustic metamaterials. First, a frequency response model of the solid structure in the ASI system was established. Subsequently, the FIP method was used to obtain the FMAM with better vibration and noise reduction performance based on the goal of maximizing the fundamental frequency of the solid structure. In particular, the constraints of the surficial acoustic pressure were considered in the optimization model of the structural fundamental frequency. Based on the deviation of the results of parametric optimization from the actual requirements, feedback adjustments were made to strengthen the parametric optimization effect. Finally, the modal acoustic characteristics of FMAM were studied using a closed acoustic box based on the ASI system. The design effect of the FIP method on the vibration and noise reduction performance of the FMAM was verified. The following conclusions were drawn:

The FMAM obtained using topology optimization increased the natural frequency (from 750 to 1210 Hz) and reduced the maximum amplitude. The change rate was also relatively large (the change rate of the first three natural frequency is 51.6%, 54.67%, and 54.79%, respectively; the change rate of the first three maximum amplitude is −22.69%, −24.54%, and −24.39%, respectively). However, the first three natural frequencies of FMAM (1026.5, 1833.4, and 1834.9 Hz, respectively) still could not avoid the mid-low frequency noise distribution range [500 Hz, 2000 Hz]. Therefore, the fundamental structural frequency required to be further improved through subsequent parametric optimization.

After parametric optimization, the fundamental frequency of the FMAM was 1662.4 Hz, an increase of 61.59%. However, a feedback adjustment was triggered due to the result of the parametric optimization being too far from the design goal. The fundamental frequency of the structure after the second optimization was 2011 Hz, and the entire modal frequency distribution interval completely avoided the mid-to low-frequency noise distribution range.

The modal acoustic experiment of the FMAM was performed using a closed acoustic box. The experiment measured that the FMAM reached a resonance state at 2010 Hz with an amplitude of 1.513 mm, consistent with the simulation results. This result illustrated the effectiveness of the FIP method in designing the fundamental frequencies of porous acoustic metamaterials.

In the future, this method can be extended to solve design problem with the mid and high frequency ranges. Moreover, the problem with multiple resonances is also worth an investigation. It is also noted that the parametric modelling is a heuristic step. Further attempts to avoid this step and automate the whole process can be regarded as a future work.

## Figures and Tables

**Table 1:** Results of former six modal analysis for porous acoustic metamaterials before and after topology optimization (TO).

Mode	Natural Frequency/Hz		Change Rate/%	Maximum Amplitude/mm		Change Rate/%
	Before TO	After TO		Before TO	After TO	
First order	677.15	1026.5	51.6	2.9615	2.2895	−22.69
Second order	1185.4	1833.4	54.67	2.8006	2.1134	−24.54
Third order	1185.4	1834.9	54.79	2.8006	2.1175	−24.39
Forth order	1554.1	2175.1	39.96	2.705	2.039	−24.62
Fifth order	1554.1	2178.9	40.2	2.705	2.0386	−24.64
Six order	1593.7	2446.4	53.5	2.6863	1.6228	−39.59

**Table 2:** The main parameters in ASI system.

**Solid**	**Young’s Modulus**	**Density**	**Poisson’s Ratio**
	2.6 GPa	1.12 g/cm^3^	0.35
Acoustic (20 °C)	Speed	Density	
	344 m/s	1.29 kg/m^3^	

**Table 3:** The value range of the main design parameters in the parametric optimization.

Design Parameters	L1 (mm)	L2 (mm)	L3 (mm)	L4 (mm)	L5 (mm)
Value range	(100, 250)	(80, 250)	(80, 250)	(3, 6)	(3, 6)
Design parameters	L6 (mm)	L7 (mm)	L8 (mm)	L9 (mm)	L10 (mm)
Value range	(2.7, 5)	(2.7, 5)	(2.5, 4.5)	(2.5, 4.5)	(2, 4)
Design parameters	L11 (mm)	L12 (mm)	L13 (mm)	Azimuth angle α1	Slope angle α2
Value range	(2, 4)	(0.5, 1.5)	(0.5, 1.5)	(0°, 45°)	(0°, 30°)

**Table 4:** Optimal values of the main design parameters.

Design Parameters	L1 (mm)	L2 (mm)	L3 (mm)	L4 (mm)	L5 (mm)
Value range	212.68	178.91	196.46	4.65	5.23
Design parameters	L6 (mm)	L7 (mm)	L8 (mm)	L9 (mm)	L10 (mm)
Value range	4.95	4.69	4.49	4.37	3.07
Design parameters	L11 (mm)	L12 (mm)	L13 (mm)	Azimuth angle α1	Slope angle α2
Value range	2.92	1.21	1.21	43.47	22.36

**Table 5:** Results of former six modal analyses for porous acoustic metamaterials before and after parametric optimization (PO).

Mode	Natural Frequency/Hz		Change Rate/%	Maximum Amplitude/mm		Change Rate/%
	Before PO	After PO		Before PO	After PO	
First order	1026.5	1662.4	61.95	2.2895	1.6459	−28.11
Second order	1833.4	2550.7	39.12	2.1134	1.3537	−35.95
Third order	1834.9	2560.1	39.52	2.1175	1.3793	−34.86
Forth order	2175.1	2868.1	31.86	2.039	1.9087	−6.39
Fifth order	2178.9	2889.5	32.61	2.0386	1.8456	−9.47
Six order	2446.4	3128.2	27.87	1.6228	1.3659	−15.83

**Table 6:** Value ranges of the optimization parameters in secondary optimization.

Design Parameters	L1 (mm)	L2 (mm)	L3 (mm)	L6 (mm)	L7 (mm)
Value range	(197, 228)	(161, 196)	(179, 214)	(4.7, 5)	(4.4, 5)
Optimal value	207.14	185.23	199.75	4.86	4.74
Design parameters	L8 (mm)	L9 (mm)	L10 (mm)	Slope angle α2	
Value range	(4.2, 4.5)	(4.1, 4.5)	(2.8, 3.3)	(19.3°, 25.4°)	
Optimal value	4.31	4.18	3.23	24.11	

## Abbreviations

FIP	Full-cycle interactive progressive
ASI systems	Acoustic-structure interaction systems
FMAM	Frequency modulation acoustic metamaterial
SM	Surrogate model
SIMP method	Solid isotropic microstructures with penalization method
SPL	Sound pressure level
TO	Topology optimization
EGO	Effective global optimization
MSG exploration	Multi-start search with geometric global exploration
EI	Expected improvement
PO	Parametric optimization
